# Experimental study on the treatment of AMD by SRB immobilized particles containing “active iron” system

**DOI:** 10.1371/journal.pone.0295616

**Published:** 2023-12-11

**Authors:** Wenbo An, Xuechun Hu, He Chen, Qiqi Wang, Yonglin Zheng, Jiahui Wang, Junzhen Di

**Affiliations:** 1 School of Civil Engineering, Liaoning Technical University, Fuxin, China; 2 School of Mining Engineering, China University of Mining and Technology, Xuzhou, China; 3 School of Mechanics and Engineering, Liaoning Technical University Fuxin, Fuxin, China; The University of Akron, UNITED STATES

## Abstract

The inhibition and toxicity of high acidity and heavy metals on sulfate-reducing bacteria in acid mine drainage (AMD) were targeted. Highly active SRB immobilized particles were prepared using SRB, warm sticker wastes (iron powders), corncobs, and Maifan stones as the main matrix materials, employing microbial immobilization technology. The repair ability and reusability of highly active immobilized particles for AMD were explored. The results indicate that the adaptability of immobilized particles to AMD varied under different initial conditions, such as pH, Mn^2+^, and SO_4_^2-^. The adsorption process of immobilized particles on Mn^2+^ follows the quasi-second-order kinetic model, suggesting that it involves both physical and chemical adsorption. The maximum adsorption capacity of immobilized particles for Mn^2+^ is 3.878 mg/g at a concentration of 2.0 mg/L and pH 6. On the other hand, the reduction process of immobilized particles on SO_4_^2-^ adheres to the first-order reaction kinetics, indicating that the reduction of SO_4_^2-^ is primarily driven by the dissimilation reduction of SRB. The maximum reduction rate of SO_4_^2-^ by immobilized particles is 94.23% at a concentration of 800 mg/L and pH 6. A layered structure with a flocculent appearance formed on the surface of the immobilized particles. The structure’s characteristics were found to be consistent with sulfate green rust (Fe^II^_4_Fe^III^_2_(OH)_12_SO_4_·8H_2_O). The chemisorption, ion exchange, dissimilation reduction, and surface complexation occurring between the matrices in the immobilized particles can enhance the alkalinity of AMD and decrease the concentration of heavy metals and sulfates. These results are expected to offer novel insights and materials for the treatment of AMD using biological immobilization technology, as well as improve our understanding of the mechanisms behind biological and abiotic enhanced synergistic decontamination.

## 1 Introduction

In the process of coal resources mining and utilization, the contact of sulfide minerals in coal seams and surrounding rocks with oxygen and water will produce a large amount of AMD [1]. AMD has the characteristics of low pH, high iron, manganese, and sulfate content, and if discharged directly without treatment, it will cause serious pollution to the ecological environment and threaten human health and safety. In China, about 3.6 billion tons of mine wastewater is discharged every year, accounting for about 1/10 of the total industrial wastewater discharge in the country, but the treatment rate is only about 4.3%. Among them, the mines in northern China discharge about 120 million tons of wastewater every year, and up to 70% of it is directly discharged without proper treatment. It is one of the most serious environmental problems in the mining industry [2,3]. Therefore, it is particularly necessary and urgent to explore and seek effective AMD treatment methods and technologies, which are important for the sustainable development of agriculture and the economy in mining areas and for safeguarding residents’ physical and mental health [4–6]. The traditional neutralization and sedimentation method and artificial wetland method for AMD treatment have the disadvantages of secondary pollution, expensive treatment costs, and large floor space [7,8]. SRB is an anaerobic microorganism commonly found in nature, which can reduce SO_4_^2-^ to S^2-^ and combine with heavy metal ions to produce insoluble sulfide precipitation in the process of growth and metabolism [9,10], thus improving the alkalinity of AMD and reducing the pollution of heavy metal ions and SO_4_^2-^, with the advantages of low cost, environmental friendliness, and high efficiency [11,12]. However, the free SRB is susceptible to the acidity of AMD, the toxicity of high concentrations of heavy metal ions, and the inhibition of dissolved oxygen, which reduces the biological activity of SRB to reduce SO_4_^2-^ by dissimilation significantly, while the energy required for the growth and metabolism of SRB is provided by an external carbon source, and these problems hinder the promotion of SRB for the treatment of acidic mine drainage [13,14].

For this reason, researchers have introduced microbial immobilization techniques [15,16], where microorganisms are physically and chemically immobilized on specific carriers to enable them to maintain their activity while being resistant to adverse environmental influences, thus improving their ability to remove pollutants [17,18]. Many researchers have tried to prepare composites by organically combining free SRB with loading materials to improve the tolerance of SRB to pH, heavy metal toxicity, and dissolved oxygen in acidic mine wastewater and to provide a carbon source for SRB [19,20], creating a suitable environment for SRB growth and thus improving the activity of SRB. In recent years, immobilization of SRB using different materials as carriers to treat acidic mine wastewater has been a popular research direction. Di, J.Z.’s team used Maifan stone and modified lignite as surface adsorbent materials in combination with SRB to treat acidic mine drainage, and all of them achieved better results due to their large specific surface area and strong adsorption properties [21–24]. Shao, L., et al determined the total organic carbon (TOC) and total carbon (TC) contents of six biomass materials, such as corn cob, sugarcane bagasse, and peanut shell, in the leaching solution within 16 d. It was found that the TOC contents of all six materials accounted for more than 90% of TC in the leaching solution, and all of them could release a large amount of organic carbon, which met the basic conditions as the cohesive carbon source materials of SRB [25]. Teclu, D., et al found that molasses was tested as a possible source of carbon for the growth of SRB [26]. An, W.B., et al, Wang, X.J., et al found that zero-valent iron (ZVI) has a facilitating effect on the reduction of SO_4_^2-^ by SRB because ZVI corrodes in acidic mine wastewater to convert H^+^ to H_2_, and the generated H_2_ provides electron donor for SRB, and the generated Fe^2+^ is the active component of various enzymes in SRB cells and can act as an activator for the enzyme catalyzing the SO_4_^2-^ reduction reaction [27–29].

Warm stickers are currently the most popular disposable on-the-go warming products worldwide. Since 2009, the warm stickers market (~US$1.6 Billion/year) in China has been developing rapidly, and the annual consumption has reached more than 800 million pieces, with the discarded warm stickers residue of about 35000 tons [30]. The discarded warm stickers contain dark brown solid waste, which mainly contains iron powder, vermiculite, activated carbon, highly absorbent resin, and other adsorbent substances [31]. Among them, the iron powder can be recycled as a cheap iron source for various water treatment reactions. While other components in the waste slag such as vermiculite, activated carbon, and highly absorbent resin are excellent adsorbent materials, which can be widely used in the adsorption treatment of a variety of sewage and wastewater. At present, warm sticker waste is basically in the state of being unregulated, unattended, and discarded directly by people as general solid waste, and there are fewer reports on the recycling technology of warm sticker waste or its secondary use as an iron source at home and abroad [32]. Li, F.R., et al. [30] used the ZVI residue (to replace the iron powder) in the warm sticker waste as an adsorbent and catalyst to remove furfural from the solution. They found that the ZVI residue has a much better adsorption effect on furfural at pH 2.0 compared with pH 6.7, and the highest furfural removal was reached at 97.5%.

Throughout the above research status, scholars have achieved a lot of effective research results in the treatment of AMD, but there are few reports on using warm sticker waste (iron powders) to activate SRB immobilized particles for AMD treatment. Whether it can ensure the supply of energy during SRB dissimilation reduction is a key issue worthy of discussion. In this study, we aimed to treat AMD with high acidity and toxicity. We prepared high-activity SRB immobilized particles using SRB, warm sticker waste (iron powder), corncob, and Maifan stone as the primary matrix materials. The production method of immobilized particles was based on microbial immobilization technology. The adsorption capacity of Mn^2+^ and the reduction capacity of SO_4_^2-^ with the highly active immobilized particles under different initial conditions of AMD (including the pH, and the concentration of SO_4_^2-^ and Mn^2+^) were analyzed. The adsorption kinetics and reduction kinetics were investigated. The mechanism of the "active iron" system composed of Fe^0^/Fe^2+^ synergistically repairing acidic mine wastewater with SRB was revealed by microscopic characterization methods of scanning electron microscope (SEM) and X-ray diffraction (XRD). This study will obtain high-activity SRB immobilized particles with high treatment efficiency, convenient use, cheap material, and strong impact resistance, and provide a scientific basis for its field application in mining areas.

## 2 Experimental materials and methods

### 2.1 Experimental materials

**Fig 1:**
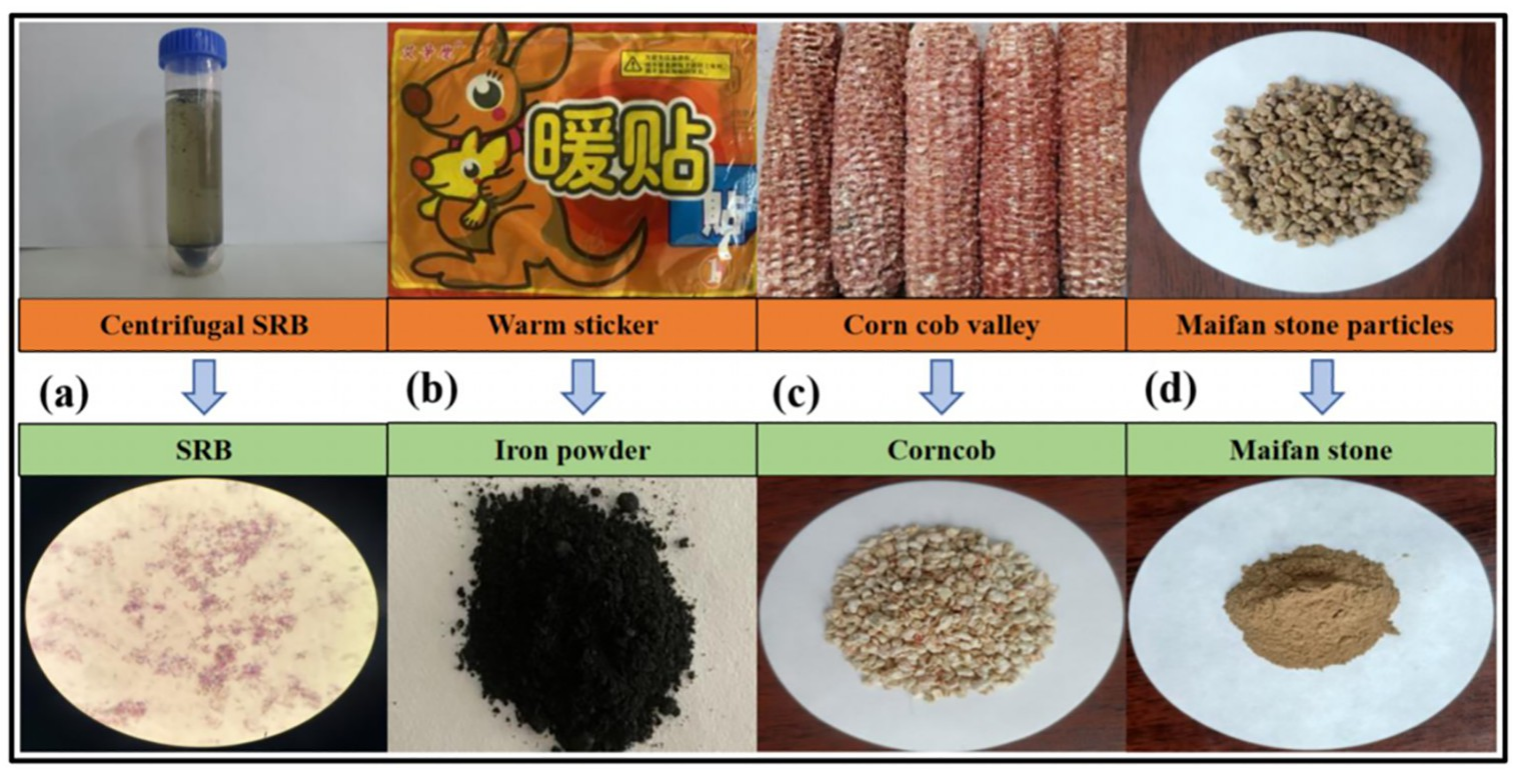
The SRB (a), iron powders (b), corncobs (c), and Maifan stones (d) used in this work.

The SRB, iron powders, corncobs, and Maifan stones used in this work are shown in Fig 1.

SRB strains come from the thick active bottom sludge from the a River in Fuxin, Liaoning Province. The impurities were filtered from sludge seed. Added an improved Starkey-type culture medium to the sludge seed and incubated it in a constant temperature anaerobic incubator at (37 ± 1) °C for two weeks until there was a strong odor of rotten eggs that could be smelled when the bottle cap was opened (caused by H_2_S). Added a small amount of culture solution dropwise to FeSO_4_ to produce black precipitates (caused by FeS). This phenomenon indicates that SRB has been successfully screened and has become the dominant strain in sludge. This conclusion is also confirmed by the results of Bao, S.H., et al [33], and Dong, Y.R., et al [34]. As shown in Fig 1a, the gram stain of the bacterial strain, the result of the gram stain is negative. After the initial identification, the bacterial of SRB. The domesticated SRB sludge suspension was centrifuged by a centrifuge at 3,000 rpm. The suspension was centrifuged 10min. The supernatant was removed and poured off after 10min. There was a concentrated sludge with a mass concentration of 500mg/L formed.

The warm sticker wastes used in the experiment came from a Chinese Herbal Medicine Co. in Hunan, China. Before the experiment, open the used warm stickers and pour out the waste residue. The main components of the warm paste residues are iron powders, vermiculites, activated carbons, highly absorbent resins, etc. The proportion of each main component is shown in Table 1.

**Table 1:** The proportion of main components in the waste residue of waste warm stickers.

The proportion of main components(wt%)					
iron powders	vermiculite	activated carbons	highly absorbent resins	inorganic salt	others
49.0	18.0	5.5	15.5	6.0	6.0

Iron powders were the reduction-activated material. The iron powders were separated from the waste residue of the warm sticker by using a magnet. The iron powders were immersion cleaned in 0.5mol/L HCl solution for 2 hours to remove their surface oxides and oil stains. The iron powders were screened into particle sizes of 48–75μm and placed in a brown ground reagent bottle.

Corncobs were the cohesive carbon source material. Corncob particles were taken from local farmland in Fuxin. The corncobs were mechanically crushed after drying, and made into particles with a diameter less than 150μm.

Maifan stones were the surface adsorption material. Maifan stones were taken from Fuxin, Liaoning Province. The Maifan stones were fully ground to a particle size of 48–75μm and washed 2~3 times with deionized water to remove the impurities and suspended matter. The Maifan stones dried at 105°C.

**Fig 2:**
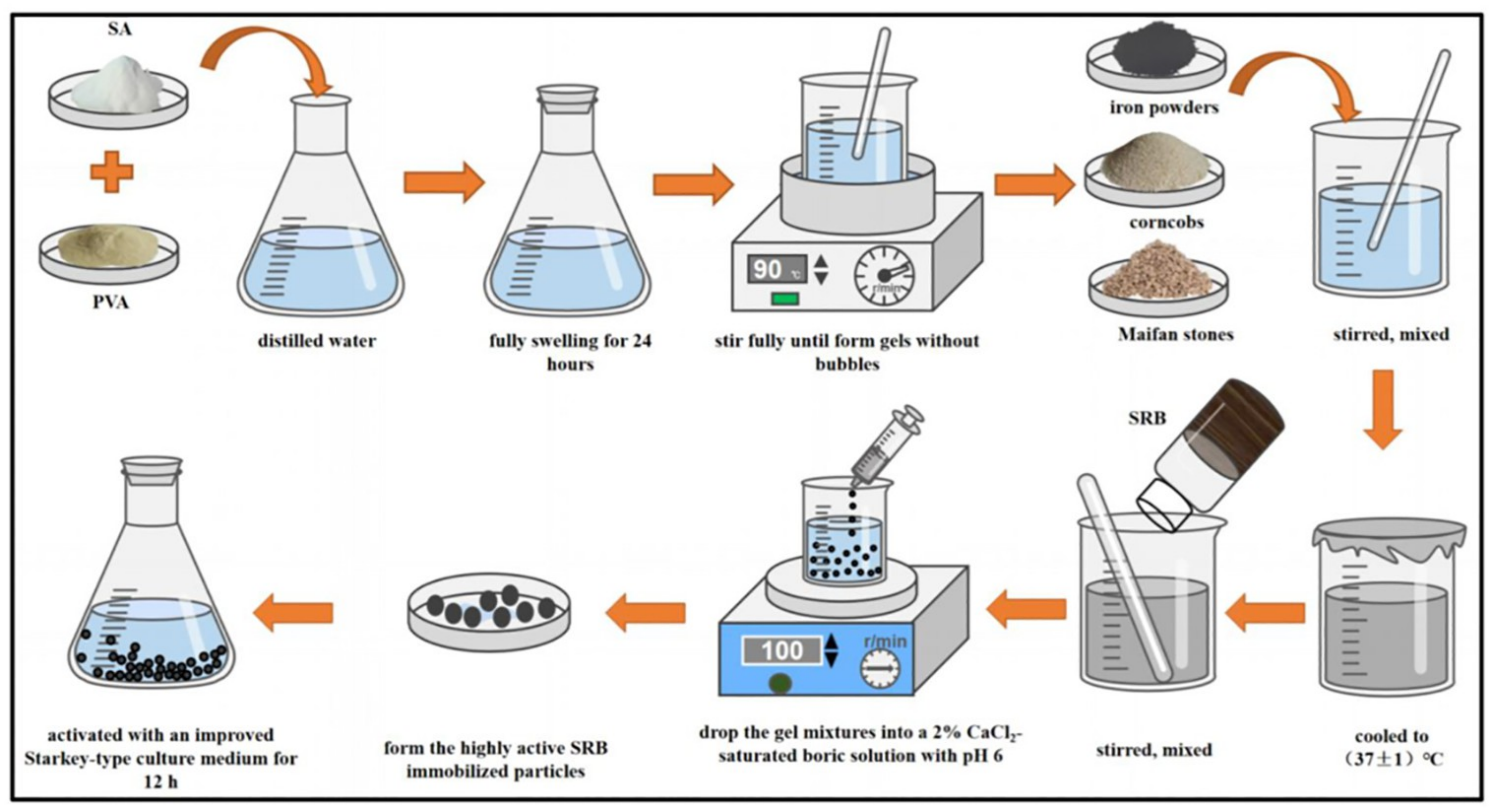
Preparation process of highly active SRB immobilized particles.

According to the previous research method of Di Junzhen’s research group [35–37], high-activity SRB immobilized particles were prepared. The production steps of highly active SRB immobilized particles were as follows. ① The polyvinyl alcohol (PVA, 10g) and sodium alginate (SA, 3g) were dissolved in 100mL distilled water and left it sealed at room temperature for 24h to ensure that they fully swelled. Then put it into a 90°C constant temperature water bath for stirring until the gels were formed without bubbles. ② The iron powders, corncobs, and Maifan stones were slowly added into the gels in turn, fully stirred, sealed, and cooled to (37 ± 1) °C. ③ The SRB sludges were cast into the prepared gels and stirred well. ④ A specific syringe was used to drop the gel mixtures into a 2% CaCl_2_-saturated boric solution with pH 6 to form immobilized particles, which were cross-linked with a hexagonal stirrer at a stirring rate of 100 rpm. ⑤ The immobilized particles were taken out after cross-linking for 4 hours. The immobilized particles were rinsed with 0.9% physiological saline, and then the surface water was sucked dry. This process was repeated three times. ⑥ The immobilized particles were activated with an improved Starkey-type culture medium without organic components for 12 h in an anaerobic environment before use. It is worth noting that the optimal dosage of iron powders, corncobs, Maifan stones, and SRB sludge in steps ② and ③ is determined according to the results of the single factor experiment and response surface experiment. The preparation process of highly active SRB immobilized particles is shown in Fig 2.

### 2.2 Simulated acid mine drainage

The monitoring data of the mine water in a mining area in Huludao, Liaoning Province over the past 5 years are shown in Table 2. Based on the table, it is evident that the mine water in this mining area is classified as typical AMD. This type of water is known for its low acidity, high levels of heavy metals, and elevated sulfate concentrations. To replicate the actual groundwater’s fluctuation and complexity, combined with the Mn^2+^, SO_4_^2-^ concentrations and pH value of AMD simulated by Dong, Y.R., et al [38]. The experimental water samples were prepared to mimic the water quality characteristics of this mine water. The concentrations of Mn^2+^, Mg^2+^, Ca^2+^ and SO_4_^2-^ in the AMD simulated in laboratory were 4 mg/L, 6 mg/L, 100 mg/L and 800 mg/L respectively, and the pH value was 4.0.

**Table 2:** Actual water quality of mine water in a mining area in Huludao (2018–2022).

Actual Water Quality of Mine Water in a Mining area in Huludao (2018–2022)									
Year	pH			Mn^2+^ (mg/L)			SO_4_^2-^ (mg/L)		
	Minimum	Mean	Maximum	Minimum	Mean	Maximum	Minimum	Mean	Maximum
2022	2.45	3.85	5.89	1.21	3.93	5.21	489	816	2050
2021	2.14	3.69	6.02	1.36	3.48	4.98	546	960	2350
2020	2.36	3.49	6.14	1.02	2.15	5.15	620	1025	2540
2019	2.11	4.03	5.33	0.95	4.25	4.25	547	846	2034
2018	2.12	4.51	5.47	1.25	3.89	4.36	523	756	2010

### 2.3 Experimental methods

#### 2.3.1 Response surface methodology experiment

Based on the results of the previous single-factor experiments, the Response Surface Methodology (RSM) is used to further optimize the experimental results. A 4-factor, 3-level response surface optimization experiment was conducted by using the Box-Behnken Design (BBD) model. There were 29 sets of RSM experiments. The four factors (three-level) of the RSM experiment were the dosage of SRB sludge (20, 30, 40 g/L), iron powders (5, 6, 7 g/L), corncobs (4, 5, 6 g/L), and Maifan stones (2, 3, 4 g/L), respectively. The response values of the RSM experiment were the release amount of chemical oxygen demand (COD), pH value, and the removal rate of Fe^2+^, Mn^2+^, and SO_4_^2-^, respectively. The RSM experiment obtained the optimal ratio of highly active SRB immobilized particles. The optimal dosage was that the SRB sludges were 30 g/L, the iron powders were 5 g/L, the corncobs were 5 g/L, and the Maifan stones were 3 g/L, respectively. At this point, the highly active SRB immobilized particles have the best treatment effect on AMD. The removal rates of the total Fe (TFe), Mn^2+^, and SO_4_^2-^ were 98.95%, 97.82%, and 83.88%, respectively. The release amount of COD was 157mg/L, and the pH was increased to 7.01.

The water quality characteristics of simulated AMD used in the experiments were pH = 4, and the mass concentrations of Fe^2+^, Mn^2+^, and SO_4_^2-^ were 10mg/L, 2.0mg/L, and 800mg/L, respectively. The water quality of treated effluent met the limits specified in the “Sanitary Standards for Drinking Water (GB5749-2006)” [39], which were that the pH limits ranging from 6.5 to 8.5, and the mass concentration limits for iron, manganese, and sulfate were 0.3mg/L, 0.1mg/L, and 250mg/L, respectively.

#### 2.3.2 The adsorption kinetics experiment of Mn^2+^

In this experiment, the highly active SRB immobilized particles (0.5 g/L) were added to the simulated AMD. The simulated AMD had different initial pH values, and different mass concentrations of Mn^2+^, and SO_4_^2-^, respectively. The beakers were sealed and placed on a constant temperature heated magnetic stirrer at 30°C and 100 rpm. The remaining concentration of Mn^2+^ in the solution was measured every 30 minutes. The adsorption mechanism of Mn^2+^ in AMD by highly active SRB immobilized particles was analyzed based on the intraparticle diffusion model, quasi-first-order kinetic model, and quasi-second-order kinetic model, respectively. The intraparticle diffusion model, the quasi-first-order kinetic model, and the quasi-second-order kinetic model are shown in Eqs (1)~(3). Three sets of parallel experiments were set up, and the average value was taken as the final value. Among them, the pH values were adjusted to 2, 4, and 6 with an H_2_SO_4_ solution. The mass concentrations of SO_4_^2-^ were adjusted to 800, 1500, and 2500 mg/L with Na_2_SO_4_ solution, and the mass concentrations of Mn^2+^ were adjusted to 1.0, 2.0, and 4.0 mg/L with MnSO_4_ solution.

$$q_{\text{t}}=K_{\text{id}}\cdot t^{0.5}+\text{C}$$ (1)

where *q*_t_ is the adsorption capacity of Mn^2+^ at a moment, mg/g. The *K*_id_ is the internal diffusion rate constant of the particles, mg/(g·h^1/2^). The C is the constant related to the thickness of the boundary layer, mg/g.

$$\text{ln}\left(q_{\text{e}}-q_{\text{t}}\right)=\text{ln}q_{\text{e}}-K_{1}\cdot t$$ (2)

$$t/q_{\text{t}}=1/K_{2}\cdot q_{\text{e}}{}^{2}+t/q_{\text{e}}$$ (3)

where *q*_e_ is the adsorption amount at adsorption equilibrium, mg/g, the *K*_1_ is the reaction rate constant of the quasi-first-order kinetic model, h^-1^, the *K*_2_ is the reaction rate constant of the quasi-second-order kinetic model, mg/(g·h).

#### 2.3.3 The reduction kinetics experiment of SO_4_^2-^

In this experiment, the highly active SRB immobilized particles (0.5 g/L) were added to the simulated AMD. The simulated AMD had different initial pH values, and different mass concentrations of Mn^2+^, and SO_4_^2-^, respectively. The beakers were sealed and placed on a constant temperature heated magnetic stirrer at 30°C and 100 rpm. The remaining concentration of SO_4_^2-^ in the solution was measured every 30 minutes. The reduction mechanism of SO_4_^2-^ in AMD by highly active SRB immobilized particles was analyzed based on zero-level and one-level reaction kinetic models. The reduction process of SO_4_^2-^ by immobilized particles can be described by the zero-order reaction kinetic model and first-order reaction kinetic model, which was shown in Eqs (4) and (5). Three sets of parallel experiments were set up, and the average value was taken as the final value. Among them, the pH values were adjusted to 2, 4, and 6 with an H_2_SO_4_ solution. The mass concentrations of SO_4_^2-^ were adjusted to 800, 1500, and 2500 mg/L with Na_2_SO_4_ solution, and the mass concentrations of Mn^2+^ were adjusted to 1.0, 2.0, and 4.0 mg/L with MnSO_4_ solution.

$$C_{\text{t}}=C_{0}-k_{0}t$$ (4)

$$lnC_{\text{t}}=lnC_{0}-k_{1}t$$ (5)

Where *C*_0_ is the initial mass concentration of SO_4_^2-^, mg/L, *C*_t_ is the mass concentration of SO_4_^2-^ at a moment, mg/L, *k*_0_ is the reaction rate constant of zero-order reaction kinetic model, mg/(L·h), *k*_1_ is the reaction rate constant of first-order reaction kinetic model, h^-1^.

#### 2.3.4 Regeneration and reuse experiments

In the regeneration experiment, the saturated immobilized particles containing Mn^2+^ and SO_4_^2-^ were rinsed with deionized water several times and dried in an oven at 80°C for 24h. The dried immobilized particles were added to the analytical solution of 100mL 1M NaOH, 0.1 M NaOH, H_2_O, 1M HCl, and 0.1 M HCl, respectively, and shaken in a shaking table at 150 rmp at 30°C. After 12h, the samples were removed by pipetting gun and filtered by 0.45 μm filter membrane. Measure the concentration of Mn^2+^ and SO_4_^2-^ in the solution and calculate the desorption rate. To explore the reusability of immobilized particles, the best eluents were selected for subsequent reuse experiments. The isolated immobilized particles were washed with deionized water to remove the eluent and dried in an oven at 50°C for 24h to determine the adsorption capacity of the recovered adsorbent for Mn^2+^ and SO_4_^2-^. After adsorption, the immobilized particles were added to the optimal eluent again, and the second adsorption-desorption cycle experiment was carried out. A total of 5 adsorption-desorption cycles were carried out in the experiment.

### 2.4 Experimental instrument and water quality testing method

The instruments used in the experiment are all owned by the Water Treatment Laboratory of the School of Civil Engineering, Liaoning Technical University. The experimental instruments are as follows: 723C visible spectrophotometer, TG-328A electronic balance, PHS-3C precision pH meter, MY3000-6M six link mixer, HZ-9811K dual speed constant temperature oscillator, 800–1 medical centrifuge, HG101-2A electric blast drying oven, ET99730 COD analyzer, HH-S12 electric constant temperature water bath, HPS-280 biochemical incubator, HD-ZK grinder, Hitachi S-3400N electron microscope scanner, XRD-6100 diffraction spectrometer.

The water quality testing methods are all carried out following national standards. The testing methods are as follows: the pH value was detected by the glass electrode method (HJ 1147–2020), the mass concentration of TFe was detected by the o-phenanthroline spectrophotometric method (HJ/T 345–2007), the mass concentration of Mn^2+^ was detected by potassium periodate spectrophotometric method (GB 11906–89), the mass concentration of SO_4_^2-^ was detected by barium chromate spectrophotometric method (HJ/T 342–2007), the release amount of COD was detected by rapid extinction spectrophotometric method (HJ/T 399–2007).

## 3 Results and discussion

### 3.1 The adsorption capacity of highly active SRB immobilized particles for Mn^2+^

**Fig 3:**
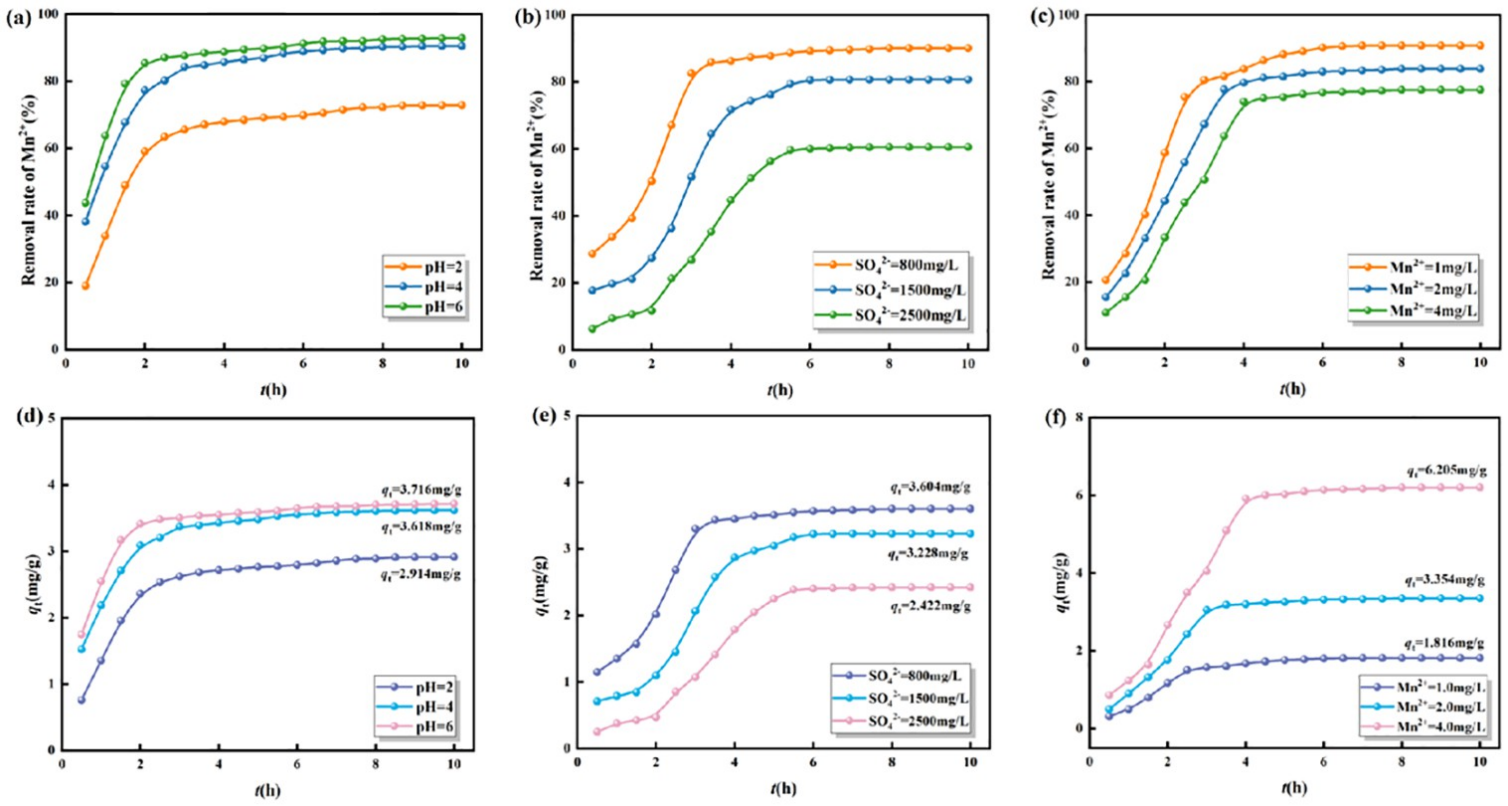
The adsorption capacity of immobilized particles for Mn^2+^ under different initial conditions. (a, d) Different initial pH values. (Mn^2+^ = 2 mg/L, SO_4_^2-^ = 800mg/L). (b, e) Different initial concentrations of SO_4_^2-^. (Mn^2+^ = 2 mg/L). (c, f) Different initial concentrations of Mn^2+^. (SO_4_^2-^ = 800mg/L).

The adsorption capacity of highly active SRB immobilized particles for Mn^2+^ was characterized by the removal rate of Mn^2+^ at a certain time. The removal rates of Mn^2+^ are shown in Fig 3a–3c. The adsorption capacity of Mn^2+^ is shown in Fig 3d–3f.

As shown in Fig 3a and 3d, the initial pH value was directly proportional to the removal rate of Mn^2+^, i.e., the lower the pH value was, the lower the adsorption efficiency of Mn^2+^ was. The "convex" curve indicated that the adsorption of Mn^2+^ by immobilized particles reacted rapidly before 1.5 hours, and the removal rate slowly increased from 1.5 to 7 hours. After 7 hours, the adsorption reaction reached equilibrium. At this time, the adsorption amounts were 2.914, 3.816, and 3.875mg/g, respectively. There are two reasons for the rapid reaction in the early stage. Firstly, SRB needs a period to adapt to the acidity and heavy metal toxicity of AMD, and its growth and metabolism are relatively slow. At this point, the highly active SRB immobilized particles have strong electrostatic adsorption and biological flocculation effects on Mn^2+^. These two effects mainly rely on the surface electronegativity of immobilized particles and the extracellular polymer secreted by immobilized particles [40]. Secondly, after a period of reaction, the Fe^0^/Fe^2+^ formed an "active iron" system inside the immobilized particles, which promoted the dissimilatory reduction of SRB. The generated S^2-^ can quickly form MnS precipitation with Mn^2+^ in AMD solution. At the same time, Maifan stones have strong adsorption properties for Mn^2+^ through the effects of ion exchange and surface complexation reactions [41]. In the middle stage of the reaction, there was a slow increase in the removal rate of Mn^2+^. The occurrence of this phenomenon is due to the gradual reduction of adsorption sites on the surface and inside of immobilized particles as the reaction progresses. The adsorption efficiency of immobilized particles gradually decreases. At the late stage of the reaction, the adsorption sites were all occupied and the immobilized particles reached the adsorption saturation state.

As shown in Fig 3b and 3e, the initial mass concentration of SO_4_^2-^ was inversely proportional to the removal rate of Mn^2+^, i.e., the lower the initial mass concentration of SO_4_^2-^ was, the higher the adsorption efficiency of Mn^2+^ was. The removal rate of Mn^2+^ by immobilized particles showed a consistent trend under different initial mass concentrations of SO_4_^2-^. The reaction was slow before 1.5 hours, and the removal rate increased rapidly from 1.5 to 6 hours. After 6 hours, the adsorption reaction reached equilibrium. At this time, the adsorption amounts were 3.904, 3.228, and 2.420mg/g, respectively. The slower reaction in the early stage was due to the increased reaction load, which inhibited the activity of SRB, and weakened the process of SRB dissimilatory reduction of sulfate ions. After a period of reaction, the activity of SRB was restored and the reaction rate of Mn^2+^ generated MnS precipitation was increased.

As shown in Fig 3c and 3f, the initial mass concentration of Mn^2+^ was inversely proportional to the removal rate of Mn^2+^, i.e., the higher the initial mass concentration of SO_4_^2-^, the lower the adsorption efficiency of Mn^2+^. The reaction was rapid in the early stage of the reaction, but the time for the adsorption reaction to reach equilibrium was different, which showed that the lower the initial mass concentration of Mn^2+^ was, the adsorption saturation of immobilized particles was reached preferentially. The adsorption saturation was 1.816 mg/g at 2.5 h for an Mn^2+^ concentration of 1.0 mg/L. The adsorption saturation was 3.854 mg/g at 3.5 h for an Mn^2+^ concentration of 2.0 mg/L. The adsorption saturation was 6.205 mg/g at 4 h for an Mn^2+^ concentration of 4.0 mg/L.

### 3.2 The adsorption kinetic process of Mn^2+^ by highly active SRB immobilized particles

The experimental data were fitted with the intra-particle diffusion model (Weber-Morris model), quasi-first-order kinetic model, and quasi-second-order kinetic model, respectively. The adsorption mechanism of Mn^2+^ by immobilized particles under different initial conditions was analyzed from the perspective of kinetics through the above kinetic models.

#### 3.2.1 Intraparticle diffusion model (Weber-Morris model)

**Fig 4:**
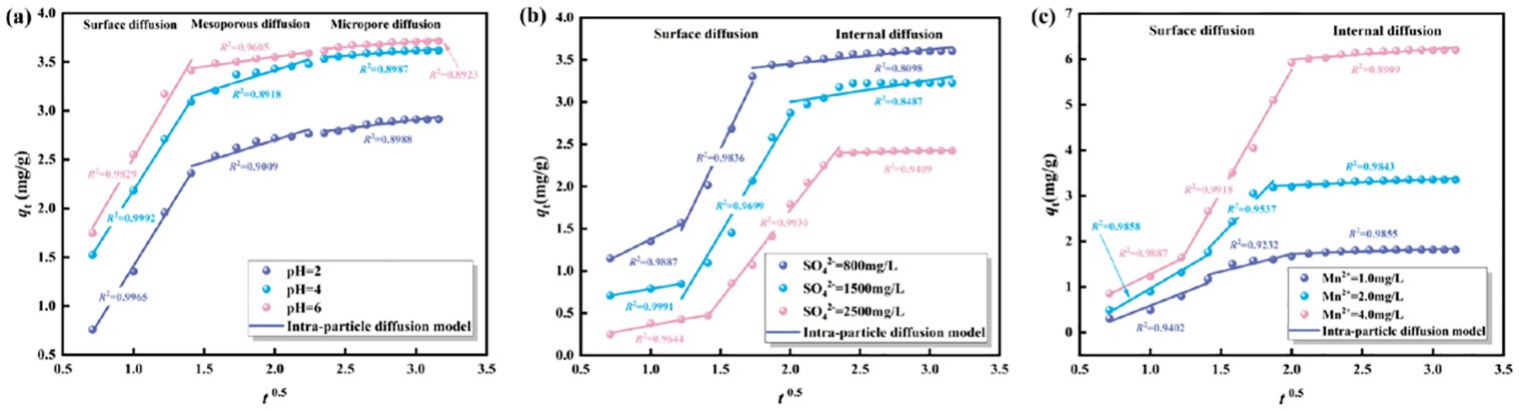
The intra-particle diffusion kinetics model of Mn^2+^ adsorbted by immobilized particles. (a) Different initial pH values. (b) Different initial concentrations of SO_4_^2-^. (c) Different initial concentrations of Mn^2+^.

The intra-particle diffusion kinetics model and adsorption parameter of Mn^2+^ adsorbed by immobilized particles are shown in Fig 4 and Table 3, respectively. As shown in Fig 4a, there were three fitting stages according to the fitting curve of the intra-particle diffusion model. In the initial stage, the adsorption rate was relatively fast, and there were a large number of active adsorption sites on the surface of immobilized particles that could adsorb Mn^2+^. At this stage, the adsorption reaction mainly involved surface adsorption, and it relied on the strong electrostatic adsorption of Mn^2+^ by the negative electrical properties on the surface of immobilized particles. In the second stage, the adsorption rate was increased slowly. The adsorption sites were gradually occupied, and a concentration gradient was formed between the surface and interior of the immobilized particles. Therefore, this phenomenon promoted the diffusion of Mn^2+^ within the mesopore of immobilized particles. In the third stage, the adsorption of Mn^2+^ by immobilized particles mainly occurred in micropores, and the adsorption rate tended to be balanced. The different slopes of the three stages showed that the adsorption of Mn^2+^ by immobilized particles was a progressive process, and the surface adsorption process was controlled by the thickness of the boundary layer. As shown in Table 3, the internal diffusion rate constant *K*_3d_ and *K*_2d_ are much smaller than *K*_1d_, it was indicated that the internal diffusion rate of Mn^2+^ increased slowly. As shown in Fig 4b and 4c, there were also three fitting stages according to the fitting curve of the intra-particle diffusion model. However, the first two stages belong to surface diffusion, and the latter stage belongs to internal diffusion. The adsorption rate of *K*_1d_ in the initial stage of surface diffusion is lower than that of *K*_2d_ because SRB activity is lower (Table 3). In addition, the fitted curve is multilinear over the entire time range and the fitted curve does not pass through the origin, it was indicated that the adsorption process was not only influenced by the internal diffusion of the particles but was also jointly controlled by the other adsorption stages [42]. The adsorption rate of Mn^2+^ by immobilized particles was determined by both boundary layer effects and external mass transfer effects [43].

**Table 3:** Intra-particle diffusion adsorption parameter of Mn^2+^ adsorbed by immobilized particles.

Parameter	Initial pH values			Initial concentration of SO_4_^2-^ (mg/L)			Initial concentration of Mn^2+^ (mg/L)		
	2	4	6	800	1500	2500	1.0	2.0	4.0
Intra-particle diffusion model *q*_t_ = *K*_id_·*t* ^0.5^+C									
*K*_1d_	2.3251	2.2572	2.4515	0.8244	0.2712	0.3072	1.2140	1.8004	1.5251
*C*_1_	-0.9135	-0.0717	0.0617	0.5515	0.5146	0.0467	-0.6227	-0.8319	-0.2422
*R*_1_^2^	0.9965	0.9992	0.9829	0.9887	0.9991	0.9644	0.9402	0.9858	0.9887
*K*_2d_	0.4544	0.4566	0.1982	3.2482	0.7686	2.1271	0.7646	3.2195	5.3568
*C*_2_	1.7922	2.5038	3.1547	-2.6965	-2.7087	-2.5330	0.1944	-2.6936	-4.9445
*R*_2_^2^	0.9009	0.8918	0.9605	0.9836	0.9699	0.9930	0.9232	0.9537	0.9918
*K*_3d_	0.1834	0.1035	0.1126	0.1718	0.2577	0.0363	0.0968	0.1334	0.2260
*C*_3_	2.3589	3.3045	3.3718	3.1078	2.4869	2.3139	1.5368	2.9637	5.5412
*R*_3_^2^	0.8988	0.8987	0.8923	0.8098	0.8487	0.9409	0.9855	0.8943	0.8909

#### 3.2.2 Quasi-first-order kinetic model and quasi-second-order kinetic model

**Fig 5:**
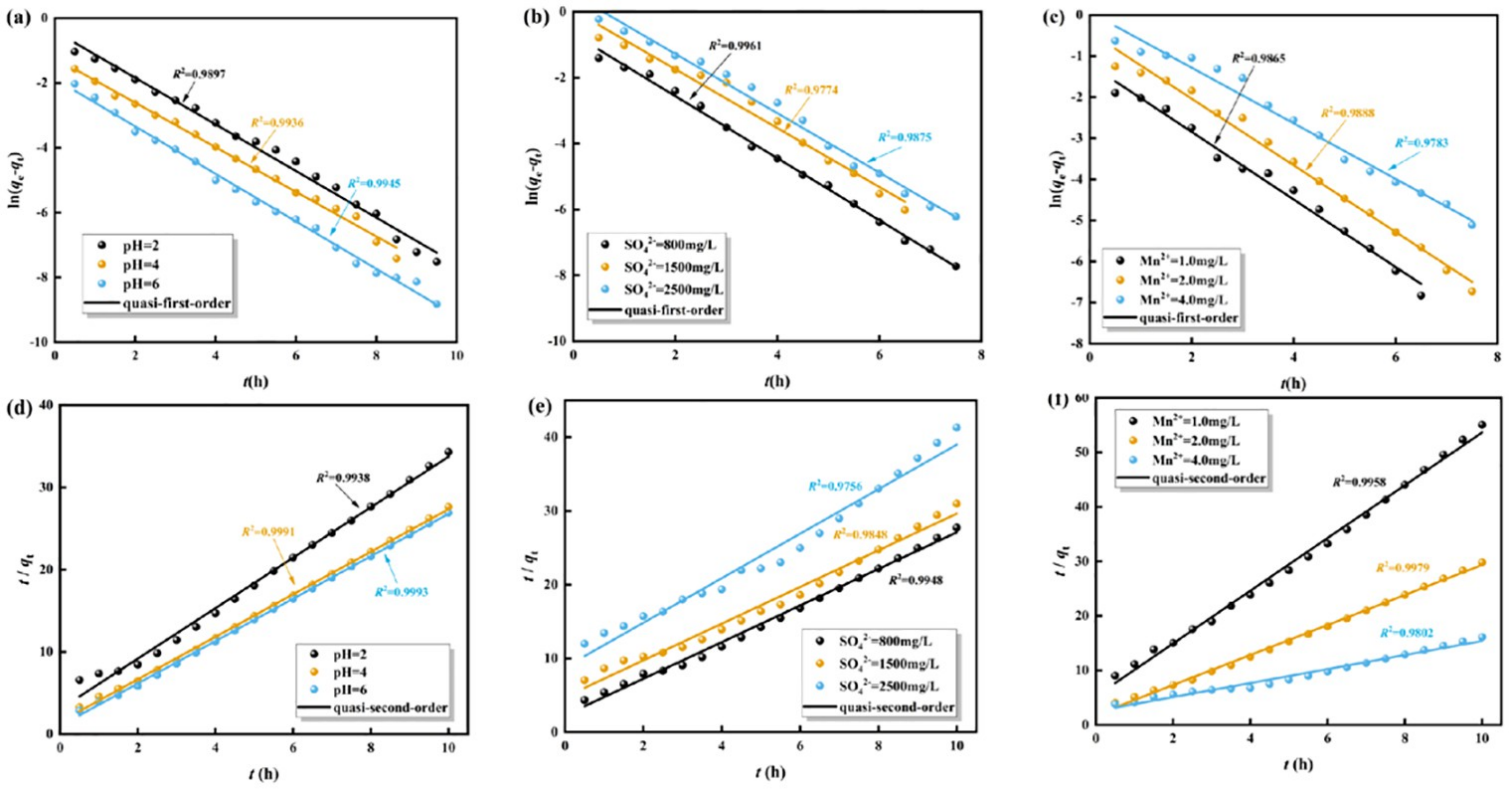
The quasi-first-order kinetic model (a-c) and quasi-second-order kinetic model (d-f) for the adsorption of Mn^2+^ by immobilized particles. (a, d) Different initial pH values. (b, e) Different initial concentrations of SO_4_^2-^. (c, f) Different initial concentrations of Mn^2+^.

The adsorption kinetics model and adsorption parameters are shown in Fig 5 and Table 4, respectively. As shown in Fig 5 and Table 4, it can be seen that the adsorption process of Mn^2+^ by immobilized particles was more consistent with the quasi-second-order kinetic model than the quasi-first-order kinetic model. The theoretical equilibrium adsorption amount *q*_e_ obtained from the quasi-second-order kinetic model was in better agreement with the experimental equilibrium adsorption amount. It can be considered that the adsorption kinetic process of Mn^2+^ by immobilized particles was most accurately described by the quasi-second-order kinetic model. The results indicate that the adsorption process was a combination of physical adsorption and chemical adsorption. The reaction process is mainly controlled by chemical reactions, rather than by material transport steps [44].

**Table 4:** Adsorption kinetic parameters of Mn^2+^ adsorbed by immobilized particles.

Parameter	Initial pH values			Initial concentration of SO_4_^2-^ (mg/L)			Initial concentration of Mn^2+^ (mg/L)		
	2	4	6	800	1500	2500	1.0	2.0	4.0
Pseudo-first-order kinetic model ln(*q*_e_-*q*_t_) = ln*q*_e_-*K*_1_*t*									
*K*_1_	0.7194	0.6882	0.7328	0.9420	0.8946	0.9014	0.8216	0.8101	0.6761
*q*_e_ (mg/g)	3.206	3.614	3.602	3.600	3.123	2.512	1.810	3.348	5.200
*R*_1_^2^_adj_	0.9897	0.9936	0.9945	0.9961	0.9774	0.9875	0.9865	0.9888	0.9783
Pseudo-second-order kinetic model *t*/*q*_t_ = 1/*K*_2_·*q*_e_^2^+*t*/*q*_e_									
*K*_2_	3.0826	4.4559	6.6115	2.7060	1.2984	1.0356	0.1326	4.1499	0.6540
*q*_e_ (mg/g)	2.908	3.869	3.878	3.928	3.320	2.412	1.856	3.834	6.272
*R*_2_^2^_adj_	0.9938	0.9991	0.9993	0.9948	0.9848	0.9756	0.9958	0.9979	0.9802

### 3.3 The reduction capacity of highly active SRB immobilized particles for SO_4_^2-^

**Fig 6:**
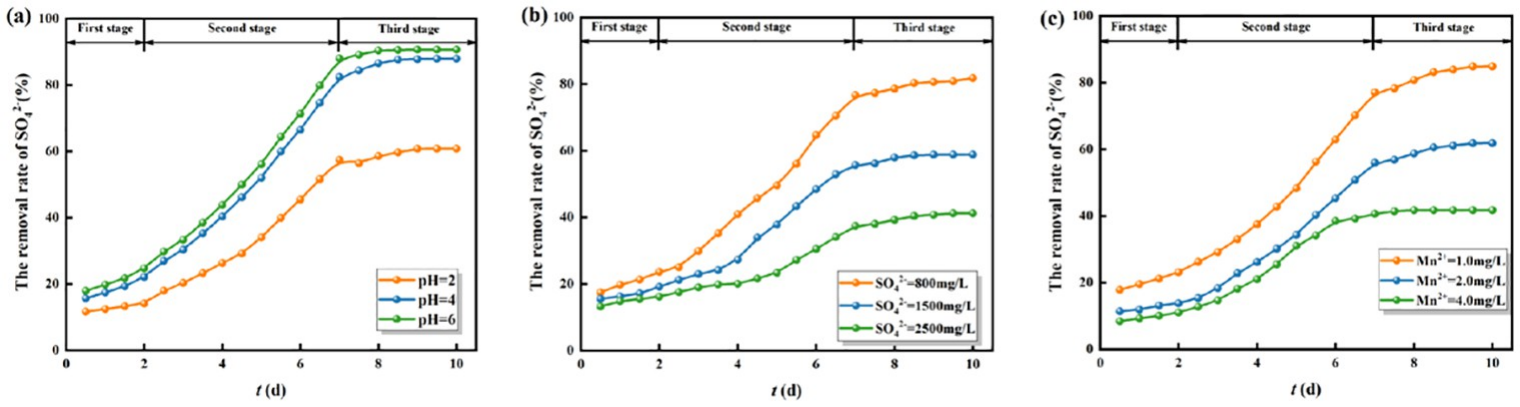
The reduction ability of immobilized particles for SO_4_^2-^ under different initial conditions. (a) Different initial pH values. (Mn^2+^ = 2 mg/L, SO_4_^2-^ = 800mg/L). (b) Different initial concentrations of SO42-. (Mn^2+^ = 2 mg/L). (c) Different initial concentrations of Mn^2+^. (SO_4_^2-^ = 800mg/L).

The reduction capacity of highly active SRB immobilized particles for SO_4_^2-^ was expressed by the removal rate of SO_4_^2-^ at a certain moment. The reduction ability of immobilized particles for SO_4_^2-^ under different initial conditions is shown in Fig 6.

As shown in Fig 6a–6c, the reduction process of SO_4_^2-^ by immobilized particles under different initial conditions showed that the lower the initial pH values, the lower the removal rate of SO_4_^2-^, and the higher the initial mass concentrations of SO_4_^2-^ and Mn^2+^, the lower the removal rate of SO_4_^2-^. The reduction process of SO_4_^2-^ is divided into three stages. In the first stage (0~2 d), the removal rate of SO_4_^2-^ was increased slowly, it was because the SRB needed to adapt to the harsh environment of acidity and heavy metal toxicity in AMD, and the metabolic process of SRB was slower. In the second stage (2~7 d), the removal rate of SO_4_^2-^ increased rapidly, it was because the trace elements released from the immobilized particles and the hydrolysis of corncobs could provide sufficient nutrients for SRB to metabolism and increase the activity of SRB. At this stage, the "active iron" system composed of F^0^/Fe^2+^ could generate a large number of H_2_ and electron donors, which could promote the dissimilatory reduction process of SO_4_^2-^ by SRB. In the third stage (after 7 d), the removal rate of SO_4_^2-^ increased slowly and stabilized, it was because although the "active iron" system composed of F^0^/Fe^2+^ could still provide electron donors for SRB, the immobilized particles were depleted of corncobs and could provide a limited carbon source for SRB, this resulting in a decrease in the activity of SRB. At the same time, the immobilized particles removed the pollutants from the solution and raised the pH value, the solution gradually became neutral. The H^+^ adsorbed by the immobilized particles gradually decreased, which provided fewer electron donors for SRB and weakened the reduction process of SO_4_^2-^. This stage finished until the SRB was inactivated and the reduction reaction of SO_4_^2-^ was stagnant. Consistent with the research of Rodrigues, C., et al. [45], H_2_S gas is produced through biological stimulation and released into the aqueous solution via SRB immobilized particles. This results in increased acidity and S^2-^ content in AMD. Further research is needed to determine whether H_2_S increases the toxicity of AMD. We hypothesize that before the SRB particles lose their activity, Fe^0^ corrosion can still consume H^+^ and reduce AMD toxicity. Additionally, the generated H_2_ can provide electric pairs for SRB dissimilation reduction, forming a continuous supply cycle. pH neutrality in AMD after the reaction can verify this conjecture.

### 3.4 The reduction kinetic process of SO_4_^2-^ by highly active SRB immobilized particles

**Fig 7:**
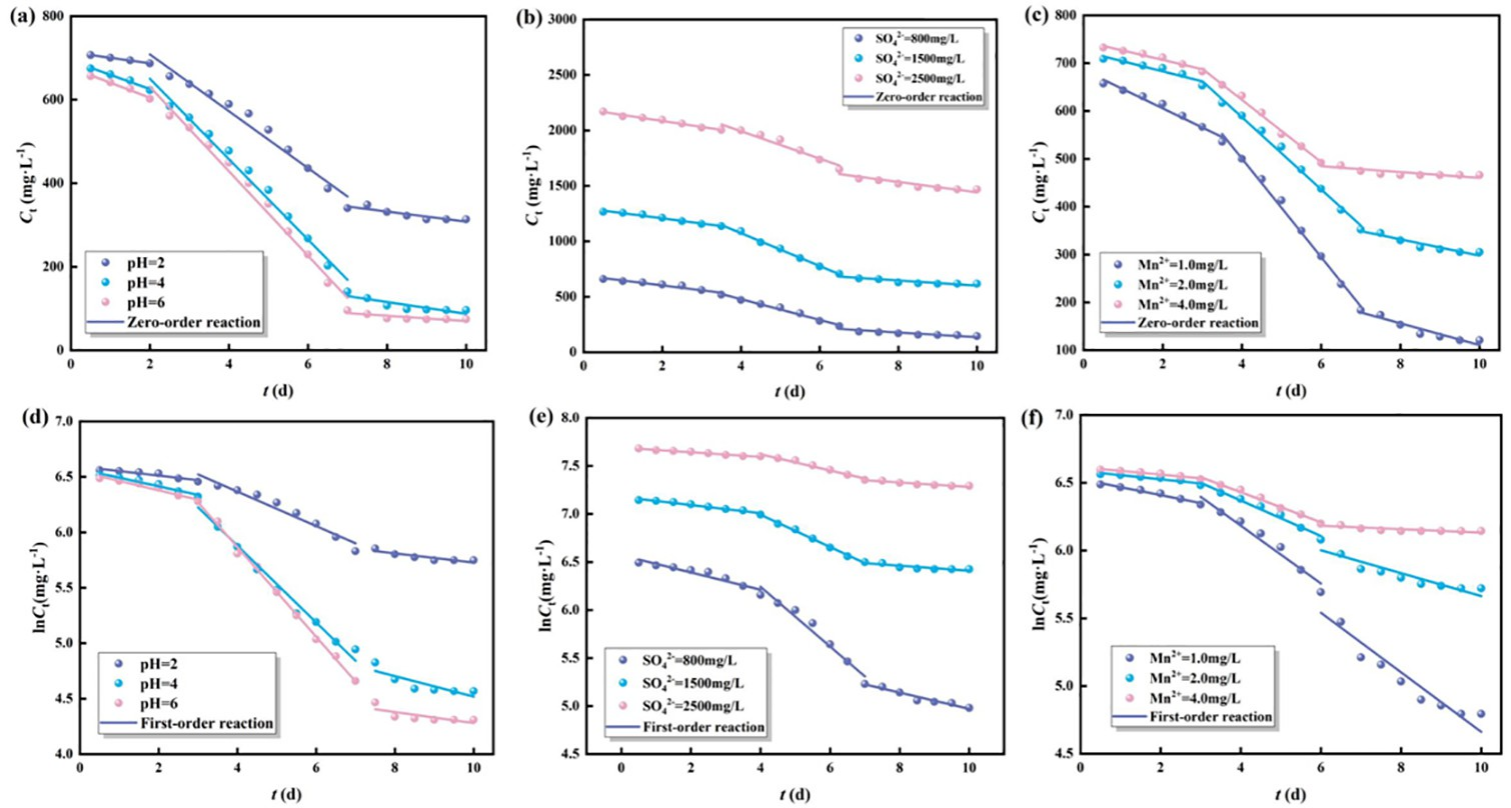
The zero-order reaction (a-c) and first-order reaction (d-f) kinetic model for the reduction of SO_4_^2-^ by immobilized particles. (a, d) Different initial pH values. (b,e) Different initial concentrations of SO_4_^2-^. (c, f) Different initial concentrations of Mn^2+^.

The reduction kinetics model and reduction parameters are shown in Fig 7 and Table 5, respectively. As shown in Fig 7 and Table 5, the reduction kinetics of SO_4_^2-^ reduction by immobilized particles under different initial conditions was more consistent with the first-order reaction kinetic model (*R*_1_^2^>*R*_0_^2^). It can be considered that the reduction kinetic process of SO_4_^2-^ by immobilized particles was mainly dominated by the dissimilatory reduction of SRB [46]. The reduction reaction of SO_4_^2-^ by immobilized particles can be carried out in two ways. One is that Maifan stones can release trace elements relying on their properties, and corncobs hydrolysis to provide the required nutrients for SRB growth and metabolism. The other is that the "active iron" system composed of F^0^/Fe^2+^ could generate large amounts of H_2_ and electron donors, which could be received by SO_4_^2-^. This process promoted the reduction of sulfate ions to sulfur ions, which could promote the synergistic effect between the immobilized particle substrates.

**Table 5:** Reduction kinetic parameters of SO_4_^2-^ adsorbed by immobilized particles.

Parameter	Initial pH values			Initial concentration of SO_4_^2-^ (mg/L)			Initial concentration of Mn^2+^ (mg/L)		
	2	4	6	800	1500	2500	1.0	2.0	4.0
Zero-order reaction kinetic model *C*_t_ = *C*_0_-*k*_0_*t*									
*K*_01_	13.232	34.030	35.730	44.030	46.457	53.884	40.0286	20.905	19.499
*C*_01_	713.600	693.600	676.280	691.245	1300.600	2193.040	685.680	724.755	745.950
*R*_01_^2^_adj_	0.9994	0.9849	0.9849	0.9388	0.9782	0.9866	0.9737	0.9137	0.9607
*K*_02_	68.033	96.252	101.064	93.568	148.060	123.430	102.968	75.429	64.939
*C*_02_	844.594	842.849	832.794	853.405	1666.230	2486.350	912.460	888.867	883.075
*R*_02_^2^_adj_	0.9724	0.9860	0.9859	0.9877	0.9964	0.9371	0.9937	0.9937	0.9939
*K*_03_	12.063	14.206	6.467	21.313	23.403	46.929	22.743	17.101	6.110
*C*_03_	428.797	229.526	134.683	349.178	834.610	1911.080	338.274	468.742	521.350
*R*_03_^2^_adj_	0.8967	0.8819	0.9028	0.8997	0.8954	0.8982	0.9220	9.2220	0.9075
*R*_0e_ (%)	60.80	87.95	90.69	81.81	58.82	41.23	84.90	61.85	41.73
First-order reaction kinetic model *lnC*_t_ *= lnC*_0_*-k*_1_*t*									
*K*_11_	0.0410	0.0770	0.0840	0.0898	0.0425	0.0240	0.0588	0.0306	0.0275
*C*_11_	6.5941	6.5703	6.5479	6.5712	7.1788	7.6915	6.5271	6.5872	6.6157
*R*_11_^2^_adj_	0.8946	0.9571	0.9563	0.9110	0.9703	0.9801	0.9731	0.9069	0.9568
*K*_12_	0.1550	0.3450	0.4030	0.3111	0.1680	0.0841	0.2130	0.1310	0.1110
*C*_12_	6.9885	7.2626	7.4812	7.4875	7.6651	7.9586	7.0367	6.8935	6.8752
*R*_12_^2^_adj_	0.9518	0.9817	0.9973	0.9624	0.9967	0.9755	0.9578	0.9802	0.9889
*K*_13_	0.0410	0.0920	0.0490	0.0847	0.0260	0.0229	0.2199	0.0846	0.0128
*C*_13_	6.1414	5.4441	4.7802	5.8186	6.6713	7.5123	6.8604	6.5103	6.2602
*R*_13_^2^_adj_	0.8193	0.9178	0.8802	0.9507	0.8865	0.9244	0.9104	0.8977	0.8099
*R*_1e_ (%)	63.50	88.57	94.23	82.38	60.57	50.23	88.69	71.43	45.26

### 3.5 Regeneration and reuse of highly active immobilized particles

**Fig 8:**
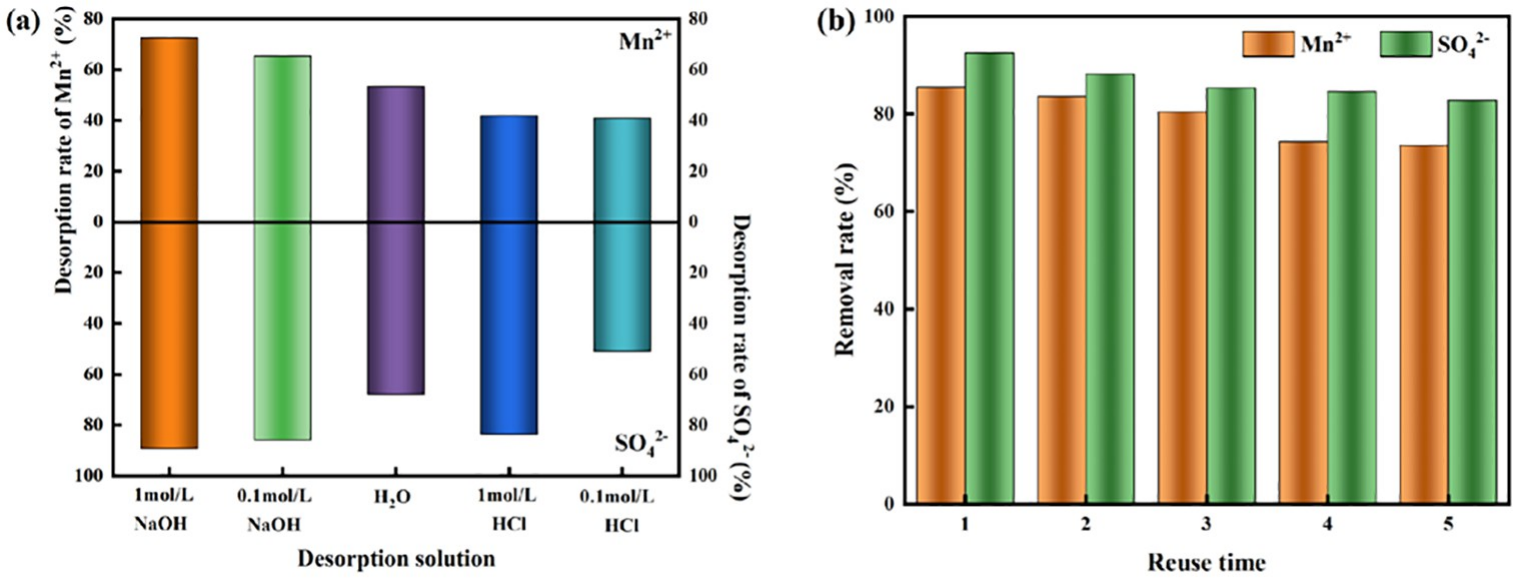
The regeneration and reuse of highly active immobilized particles. (a) regeneration. (b) reuse.

It can be seen from Fig 8a that the desorption rate of Mn^2+^ depends on the pH of the solution, and the alkaline desorption solution is conducive to the desorption of Mn^2+^. When the saturated immobilized particles are adsorbed in 0.1 mol/L NaOH solution, the maximum desorption rate of Mn^2+^ is 72.54%. It is because, in alkaline conditions, Mn^2+^ can combine with OH^-^ to form Mn(OH)_2_, which leads to an increase in the desorption rate of Mn^2+^. The desorption effect of SO_4_^2-^ in acidic and alkaline solutions is better, and the desorption rate in distilled water is the lowest. When the saturated immobilized particles were adsorbed in 0.1 mol/L NaOH solution, the maximum desorption rate of SO_4_^2-^ was 89.06%. It may be because when the solution is alkaline, more OH^-^ and SO_4_^2-^ in the solution have a competitive adsorption relationship to the adsorption sites on the surface of the immobilized particles. In 0.1 mol/L HCl solution, the desorption rate of SO_4_^2-^ is 83.53%. It may be because under acidic conditions, the desorbed SO_4_^2-^ can combine with H^+^ to form chemically stable H_2_SO_4_, which will not be attracted by the immobilized particles by electrostatic force.

Adsorption-desorption experiments were performed to evaluate the reusability of the prepared adsorbents, and 0.1 mol/L NaOH solution was used for the regeneration of adsorbed saturated immobilized particles. It can be seen from Fig 8b, that the removal rate of Mn^2+^ can still reach more than 70% after the 5th adsorption-desorption, the adsorption capacity is higher than 2.8 mg /g, and the reduction rate of SO_4_^2-^ can still reach more than 80% after the 5th adsorption-desorption. After repeated adsorption-desorption, it was found that the immobilized particles had higher reusability, higher removal rates of Mn^2+^ and SO_4_^2-^, and a certain reuse value.

In this study, the adsorption capacity of heavy metals and the reduction capacity of sulfate by highly active SRB immobilized particles in AMD were compared with some other adsorbents, as shown in Table 6. There may be some deviations between the experimental results due to the existence of different experimental conditions, but the maximum adsorption capacity of Mn^2+^ and reduction capacity of SO_4_^2-^ by immobilized particles used in this experiment clearly showed good results for AMD. By applying highly active SRB immobilized particles to AMD treatment, we cannot only solve the problem of heavy metal pollution in acidic water but also find new ways the use warm sticker residue. Moreover, the highly active SRB immobilized particles used in this study could be widely collected and had a certain reuse value. It has both economic and environmental benefits and the potential for large-scale commercial application.

**Table 6:** Comparison between various adsorbents used for AMD.

Adsorbents	Pollutants	Removal results	References
SRB combined with gangue	Fe, Mn, Sulfate	98.70%, 79.97%, 84.41%	[34]
Maifan stone-SRB-immobilized particles	Fe, Mn, Sulfate	90.51%, 85.75%, 93.61%	[23]
Shrimp shell and SRB	Fe, Al, Mn, Sulfate	99.04%, 98.47%, 100%, 99.75%	[16]
Waste pyrolysis ash	Zn, Cu, Mn, Fe, Pb, Cd	0.425, 0.593, 0.498, 18.519, 0.055, 0.039 mg/g	[47]
Attapulgite	Cu, Fe, Co, Ni, Mn	0.0053, 0.01, 0.0044, 0.0053, 0.0019 mg/g	[48]
Highly active SRB immobilized particles	Mn (pH = 2, 4, 6)	2.914, 3.618, 3.716 mg/g	This work

### 3.6 The repair mechanism of highly active SRB immobilized particles on AMD

#### 3.6.1 The results of SEM

**Fig 9:**
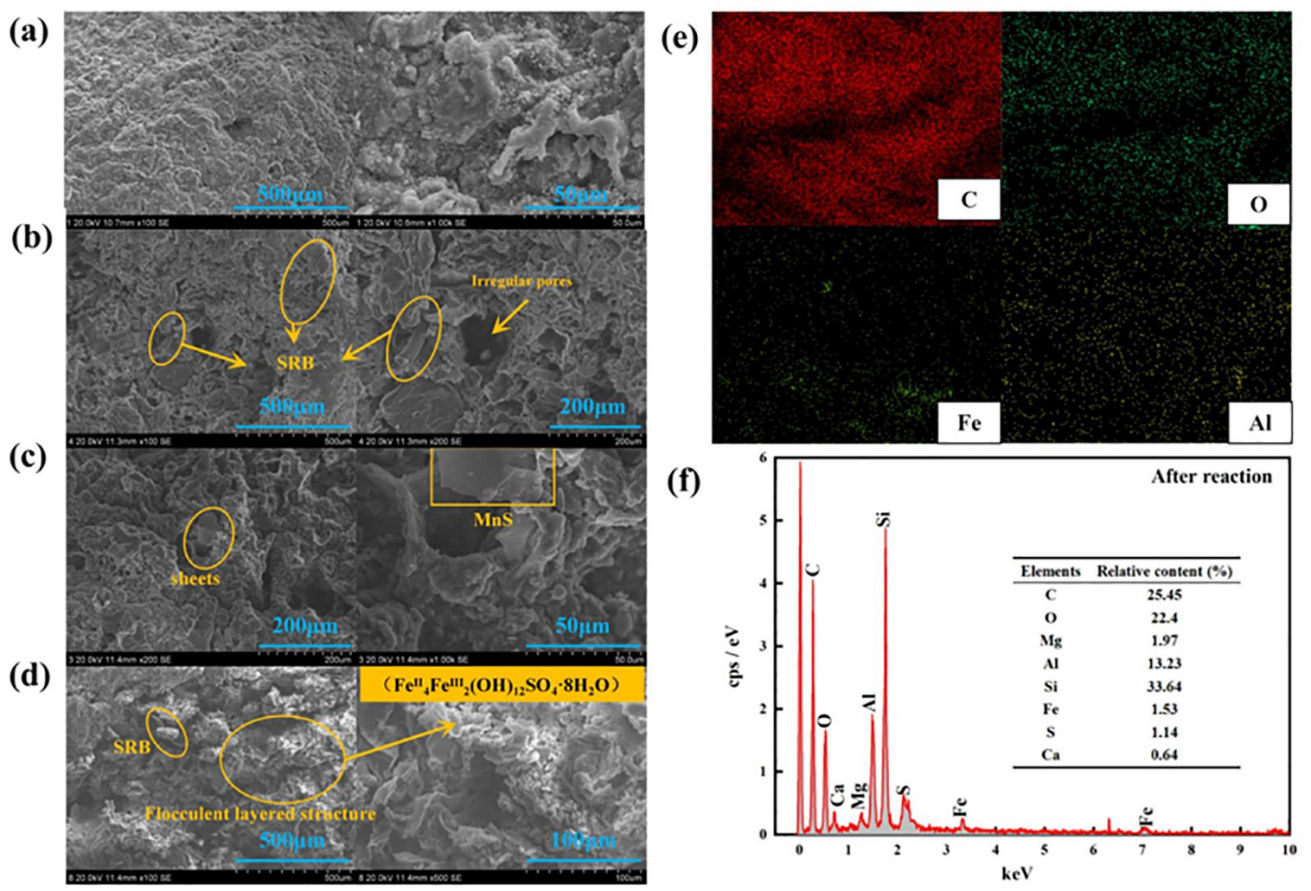
The results of scanning electron microscope on highly active SRB immobilized particles. (a) before repaired AMD. (b) SRB strains appeared around the pores. (c) form MnS precipitation. (d) sulfate green rusts. (e) element distribution after reaction. (f) elemental content after reaction.

It can be seen that the surface of immobilized particles was smooth and uniform in texture before repairing AMD (Fig 9a). After repaired AMD (taking the AMD with pH = 4, SO_4_^2-^ = 800mg/L, and Mn^2+^ = 2.0mg/L as an example), the surface of immobilized particles was rough, the pore structure was irregular, and there were some SRB strains appeared around the pores (Fig 9b). There were some sheet structural materials that appeared around the pores of immobilized particles (Fig 9c), which was consistent with the structural characteristics of MnS. The S^2-^ generated by SRB dissimilatory reduction of SO_4_^2-^ reacted with heavy metal Mn^2+^ in AMD to form MnS precipitation, and at the same time, the H_2_S and H_2_ generated would penetrate the immobilized particles have internal to external diffusion, resulting in the increase of particle pores and the leakage of the matrix inside the particles. Some flocculent layered structure materials appeared inside the particles, which was consistent with the structural characteristics of sulfate green rusts (Fe^II^_4_Fe^III^_2_(OH)_12_SO_4_·8H_2_O) [49], i.e., sulfate green rusts were the layered hydroxide based on Fe (Fe^2+^ and Fe^3+^) cations with the structural characteristics of hydrotalcite-type layered bimetallic hydroxide [50] (Fig 9d). When Fe^0^ was present in an anaerobic environment, it would react with H^+^ in the AMD to release Fe^2+^, which increased the relative content of OH^-^ in the wastewater, and this reaction would facilitate the generation of iron hydroxides, such as Fe(OH)^2+^ and Fe(OH)^+^. These iron hydroxides would react with SO_4_^2-^ in AMD to form sulfate green rusts. It can be seen from Fig 9e and 9f that the presence of C, O, S, Fe, Al, and other elements further proves the presence of matrix materials and sulfate green rust in the immobilized particles.

#### 3.6.2 The results of XRD

**Fig 10:**
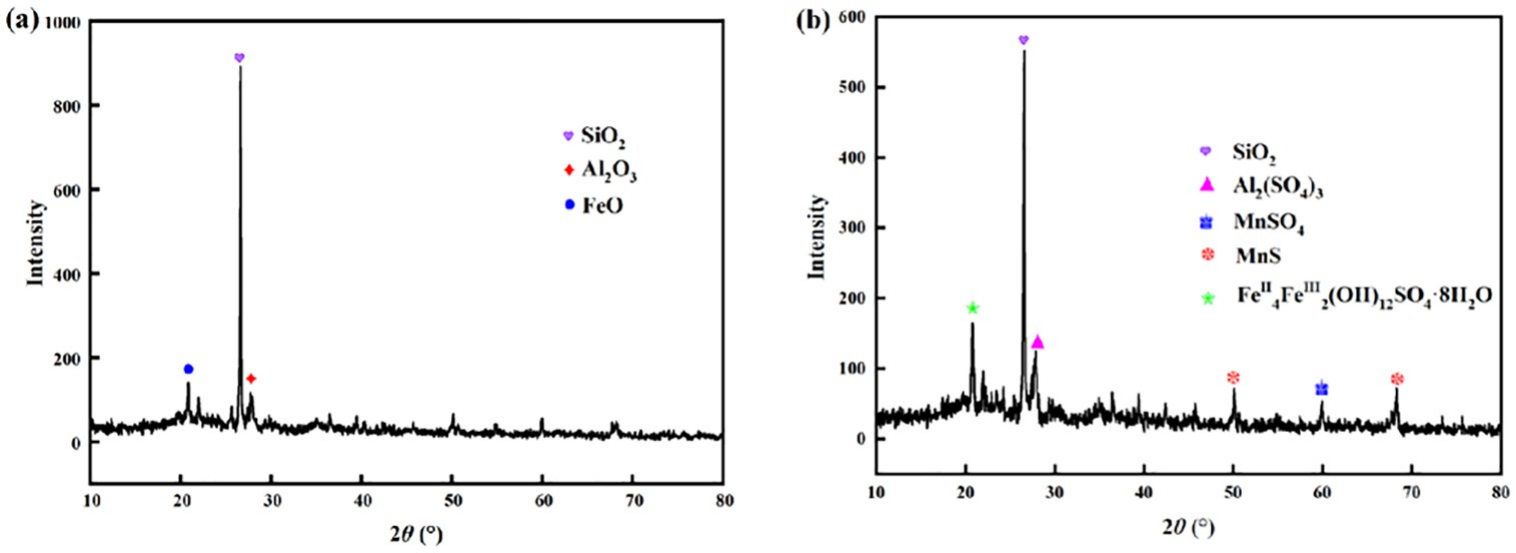
The results of X-ray diffraction (XRD) on highly active SRB immobilized particles. (a) before repaired AMD. (b) after repaired AMD.

It can be seen that the chemical composition inside the immobilized particles was changed before and after the repair of the AMD. As shown in Fig 10a, there are some chemical compounds (such as SiO_2_, Al_2_O_3_, FeO, and other components) were detected in the immobilized particles before repaired AMD, which mainly originated from the internal matrix materials of the immobilized particles (e.g. Maifan stones, iron powders, etc.). As shown in Fig 10b, there are some new chemical compounds (such as Al_2_(SO_4_)_3_、MnSO_4_, MnS, and Fe^II^_4_Fe^III^_2_(OH)_12_SO_4_·8H_2_O, etc.) were detected in the immobilized particles after repaired AMD (taking the AMD with pH = 4, SO_4_^2-^ = 800mg/L, and Mn^2+^ = 2.0mg/L as an example). these new chemical compounds mainly come from the characteristic substances produced by the adsorption, complexation, and ion exchange interaction between the matrix materials in the immobilized particles and the pollutants in AMD. The X-ray diffraction results once again confirmed that the immobilized particles can effectively remove Mn^2+^ and SO_4_^2-^ from AMD, and improve the pH value of AMD.

#### 3.6.3 The repairing mechanism of AMD by highly active SRB immobilized particles

**Fig 11:**
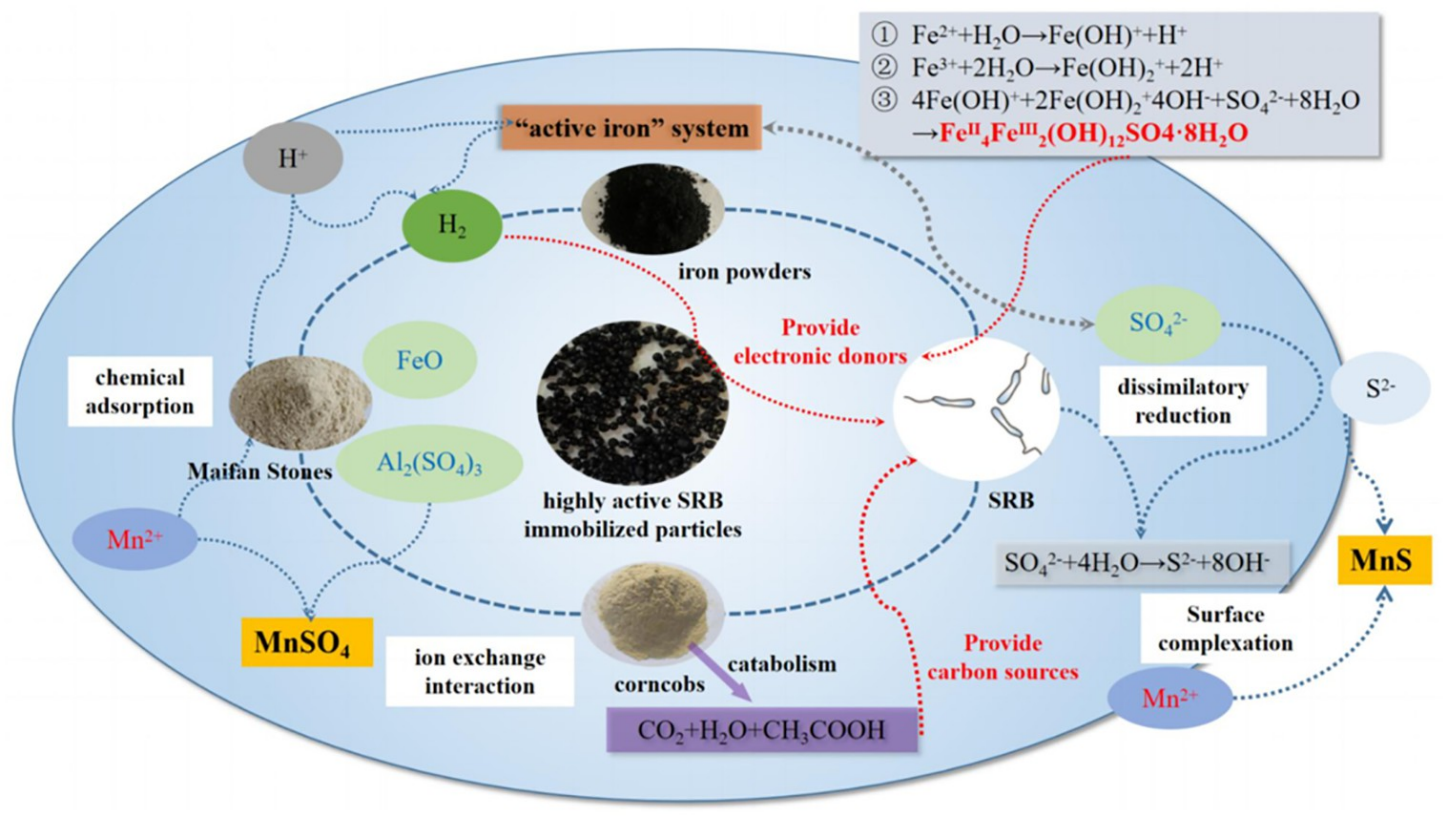
Schematic diagram of the repairing mechanism of AMD by highly active SRB immobilized particles.

The iron powders, Maifan stones, corncobs, and SRB were matrix materials of the highly active SRB immobilized particles, and the interaction between them in the AMD repaired process was described in Fig 11. The iron powders existed in the form of an "active iron" system composed of Fe^0^/Fe^2+^ in an acidic and anaerobic environment, and they have two functions. One is that they can directly react with SO_4_^2-^ to form sulfate green rust. Sulfate green rusts have a unique structure with overlapping positive and negative charge layers and contain a large amount of bound-state divalent iron. They have high chemical reaction activity and strong electron-donating ability and can be used as an electron donor for SRB. The other one is that the iron powders can react with H^+^ to form H_2_, which can also provide an electron donor for SRB dissimilatory reduction of SO_4_^2-^. Maifan stones have a loose and porous structure, a large surface area, and strong chemical adsorption. They can dissolve trace elements such as Al and Fe in an aqueous environment, which can have ion exchange interaction with Mn. At the same time, there was a large amount of Al_2_O_3_ in Maifan stones, which existed in the form of Al(OH)_2_^+^ and H_2_AlO_3_^-^ in acidic and alkaline solutions, respectively, and they have bi-directional acid-base regulation. They can reduce the content and acidity of heavy metal pollutants in AMD and weaken its toxic effect on SRB. The corncobs can decompose the nutrients required for SRB growth and metabolism and can provide the carbon source for the SRB dissimilatory reduction process. The S^2-^ generated by the SRB dissimilatory reduction process undergoes surface complexation with Mn^2+^ on the surface of immobilized particles and generated MnS. Therefore, the synergistic effect of various matrices can enhance the alkalinity of AMD and reduce the concentration of heavy metals and sulfates.

## 4 Conclusion

As an efficient adsorbent, highly active SRB immobilized particles can effectively repair AMD. It can adapt to a wide range of AMD, specifically pH levels ranging from 2 to 6, initial concentration of SO_4_^2-^ ranging from 800 to 2500 mg/L, and initial concentration of Mn^2+^ ranging from 1 to 4mg/L. The adsorption of Mn^2+^ by highly active SRB immobilized particles is a combination of physical adsorption and chemisorption. The reaction process is mainly controlled by the chemical reaction, rather than by the material transport step. The kinetic process of SO_4_^2-^ reduction by immobilized particles is mainly the dissimilation reduction of SRB. In addition, there was chemisorption, ion exchange, dissimilation reduction, and surface complexation between the matrices in the highly active SRB immobilized particles. The "active iron" system composed of Fe^0^/Fe^2+^ can ensure the energy supply in the process of SRB dissimilation reduction. The immobilized particles can improve the alkalinity of AMD and reduce the concentration of heavy metals and sulfates. The treated effluent water quality met the limits specified in the Sanitary Standards for Drinking Water (GB5749-2006).

## Supporting information

S1 File (XLSX)
